# Structure-Based Virtual Screening Allows the Identification of Efficient Modulators of E-Cadherin-Mediated Cell–Cell Adhesion

**DOI:** 10.3390/ijms20143404

**Published:** 2019-07-11

**Authors:** Andrea Dalle Vedove, Federico Falchi, Stefano Donini, Aurelie Dobric, Sebastien Germain, Giovanni Paolo Di Martino, Tommaso Prosdocimi, Chiara Vettraino, Archimede Torretta, Andrea Cavalli, Veronique Rigot, Frederic André, Emilio Parisini

**Affiliations:** 1 Center for Nano Science and Technology @PoliMi, Istituto Italiano di Tecnologia, Via Pascoli 70/3, 20133 Milano, Italy; 2 Computational Sciences, Istituto Italiano di Tecnologia, via Morego 30, 16163 Genova, Italy; 3 Department of Pharmacy and Biotechnology, University of Bologna, via Belmeloro 6, 40121 Bologna, Italy; 4 Aix Marseille Univ, CNRS, INSERM, Institut Paoli-Calmettes, CRCM, 13273 Marseille CEDEX 09, France

**Keywords:** E-cadherin, P-cadherin, cell adhesion, cell invasion, inhibitor, structure-based virtual screening

## Abstract

Cadherins are a large family of transmembrane calcium-dependent cell adhesion proteins that orchestrate adherens junction formation and are crucially involved in tissue morphogenesis. Due to their important role in cancer development and metastasis, cadherins can be considered attractive targets for drug discovery. A recent crystal structure of the complex of a cadherin extracellular portion and a small molecule inhibitor allowed the identification of a druggable interface, thus providing a viable strategy for the design of cadherin dimerization modulators. Here, we report on a structure-based virtual screening approach that led to the identification of efficient and selective modulators of E-cadherin-mediated cell–cell adhesion. Of all the putative inhibitors that were identified and experimentally tested by cell adhesion assays using human pancreatic tumor BxPC-3 cells expressing both E-cadherin and P-cadherin, two compounds turned out to be effective in inhibiting stable cell–cell adhesion at micromolar concentrations. Moreover, at the same concentrations, one of them also showed anti-invasive properties in cell invasion assays. These results will allow further development of novel and selective cadherin-mediated cell–cell adhesion modulators for the treatment of a variety of cadherin-expressing solid tumors and for improving the efficiency of drug delivery across biological barriers.

## 1. Introduction

Cadherins are transmembrane calcium-dependent molecules that mediate cell–cell adhesion through a concerted dimerization and oligomerization mechanism whereby proteins protruding from opposing cells interact with each other and form an extensive adhesive network at the cellular adherens junctions. Altered expression profiles of epithelial E-cadherin (CDH1) and neuronal N-cadherin (CDH2) have often been observed in cancer cells, most notably in the context of the epithelial-to-mesenchymal transition (EMT) process that occurs during cancer progression [1,2]. Interestingly, while E-cadherin is down-regulated in the majority of carcinomas, some epithelial ovarian cancer (EOC) cells are characterized by high expression levels of E-cadherin, which facilitates proliferation [3]. More recently, the aberrant expression of P-cadherin (CDH3) and cadherin-11 (CDH11) have also been described in the context of different types of cancer such as in malignant melanoma, breast, gastric, lung, colorectal, and pancreatic cancer [4,5,6,7,8,9]. Furthermore, cadherin-11 is a therapeutic target in rheumatoid arthritis (RA) patients [10,11]. Therefore, some cadherin family members represent interesting pharmaceutical targets in a variety of pathological conditions. As a result, an increasing number of studies aimed at the identification of potential therapeutic strategies against different cadherin family members are now being reported in the literature [12,13,14].

Despite the specific cell type localization of the different cadherin family members, all classical cadherins share a high degree of sequence homology and structural similarity. They comprise an elongated extracellular portion formed by five immunoglobulin-like extracellular cadherin domains (ECs) and an intracellular portion that, through its dynamic association with α- and β-catenin, allows communication between surface-bound cadherins and the actin cytoskeleton [15].

**Figure 1:**
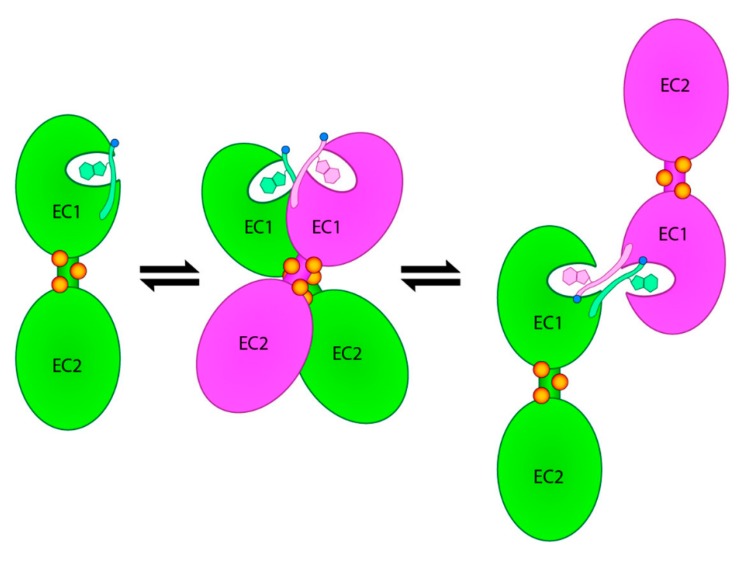
Schematic representation of the dynamic dimerization mechanism that leads from monomeric cadherin (left) to the strand-swap dimer, which involves mutual insertion of the Trp2 side chain in the binding pocket of the partner molecule (right) and back. The cadherin dimerization trajectory features a crucial intermediate configuration that is referred to as the X-dimer (center). Calcium ions are shown as orange dots.

Over the years, mutational, structural, and other biophysical studies have provided a rather detailed picture of the highly dynamic cadherin homo-dimerization mechanism that mediates the mutual recognition and binding of cadherin molecules protruding from the surface of two neighboring cells [16,17,18,19,20,21,22,23,24]. The process involves several critical steps, which are schematically shown in Figure 1.

As classical cadherins shuttle between the two ends of their dimerization trajectory, the closed monomeric conformation and the open strand-swap dimer conformation, respectively, they go through a crucial intermediate state that is commonly referred to as the X-dimer. This weakly adhesive dimeric conformation brings the adhesion arms of two interacting cadherins in close proximity and promotes strand-swap dimer formation [25]. While some crucial differences exist, most notably between type I and type II cadherins [26], this general homo-dimerization multistep mechanism is shared by all members of the classical cadherin family. A rational approach to the design of small molecule inhibitors of cadherin homo-dimerization has, so far, been hampered by the cadherin’s intrinsic dynamic behavior and by the relatively featureless nature of its dimerization interface.

Recently, we reported the crystal structure of the complex between a cadherin extracellular fragment, the human E-cadherin-EC1EC2 portion, and a small molecule, the peptidomimetic inhibitor FR159 (PDB code: 4ZTE) [27]. This high-resolution structure, which is the first and, to date, the only complex of a cadherin extracellular portion and a small molecule inhibitor, allowed the identification of a druggable interface and provided clear evidence of a possible mechanism to modulate cadherin dimerization. Publication of the structure followed an earlier study where the compound FR159 had been identified among a panel of several peptidomimetic compounds that were tested and compared in ELISA and cell adhesion assays for their ability to modulate cadherin-mediated cell–cell adhesion [28]. In this study, FR159 had been shown to partially inhibit cadherin-mediated cell adhesion at 1 mM concentration, i.e., better than ADH-1 (Exherin), a small cyclic peptide that has entered clinical trials in cancer patients (ClinicalTrials.gov Identifier: NCT00225550, NCT00264433, NCT00390676, NCT00265057, NCT00421811, and NCT01825603) [29,30,31]. In the crystal structure, the FR159 ligand was found to bind across two interacting cadherin molecules in the X-dimer conformation, forming crucial contacts with the diproline motif of their adhesion arm, a motif that has been extensively described in the literature for its crucial role in the cadherin activation mechanism. The peptidomimetic ligand is mostly stabilized by hydrophobic contacts such as, for instance, those involving the central moiety of the ligand (in particular, its phenyl ring) and the side chains of residues Ile4, Pro5, Ile7, Leu21, and Val22 from both cadherin molecules. Interestingly, the hydrophobic cavity formed by the two cadherin molecules in the X-dimer conformation is totally symmetric, as it involves the same set of residues from the two interacting proteins, and, moreover, the residues involved in ligand stabilization are conserved across most type I classical cadherins.

Here, based on this novel and unique crystal structure, we conducted a virtual screening (VS) analysis to identify putative modulators of cadherin adhesion within commercial databases of drug-like molecules. Then, by conducting cell–cell adhesion assays using human pancreatic tumor BxPC-3 cells expressing both E-cadherin and P-cadherin, we tested a number of these candidate compounds for their ability to disrupt cadherin homophilic interaction and dimerization at different concentrations. Moreover, we conducted 3D invasion assays to test the anti-invasive properties of these compounds for pancreatic cancer cells. We used RNA interference to investigate possible selectivity issues of the different ligands for either E-cadherin or P-cadherin.

## 2. Results

In our high-throughput docking (HTPD) screening, two sets of commercially available compounds were docked to the crystal structure of human E-cadherin-EC1EC2 in X-dimer conformation, as derived from the E-cadherin-FR159 complex (PDB code: 4ZTE) [27]. Prior to that, using molecular dynamics simulations, we partially rebuilt the protein in order to reintroduce the two N-terminal residues that were removed in the construct that led to the crystal structure.

First, we inspected the 1000 best-scored compounds visually. Then, based on a similarity cluster analysis, we reduced this initial pool to 200 candidate compounds, and finally we selected 15 of them to be tested experimentally, as representative of each obtained cluster.

### 2.1. Cell Adhesion

To investigate the impact of our virtual screening-derived library of small molecules on cadherin-dependent cell–cell adhesion, we analyzed whether our hits were able to counteract the capacity of BxPC-3 E-cadh/P-cadh cells to form compact spheroids when cultured in suspension. Using an inhibitor concentration of 1 mM, i.e., the lower active concentration determined for the FR159 ligand [28], we found that 5 of the 15 identified compounds (AS2, AS8, AS9, AS11, and LC11) affected BxPC-3 E-cadh/P-cadh cell–cell adhesion (see Table 1 for commercial codes and chemical formulas), although some of them showed solubility problems.

**Figure 2:**
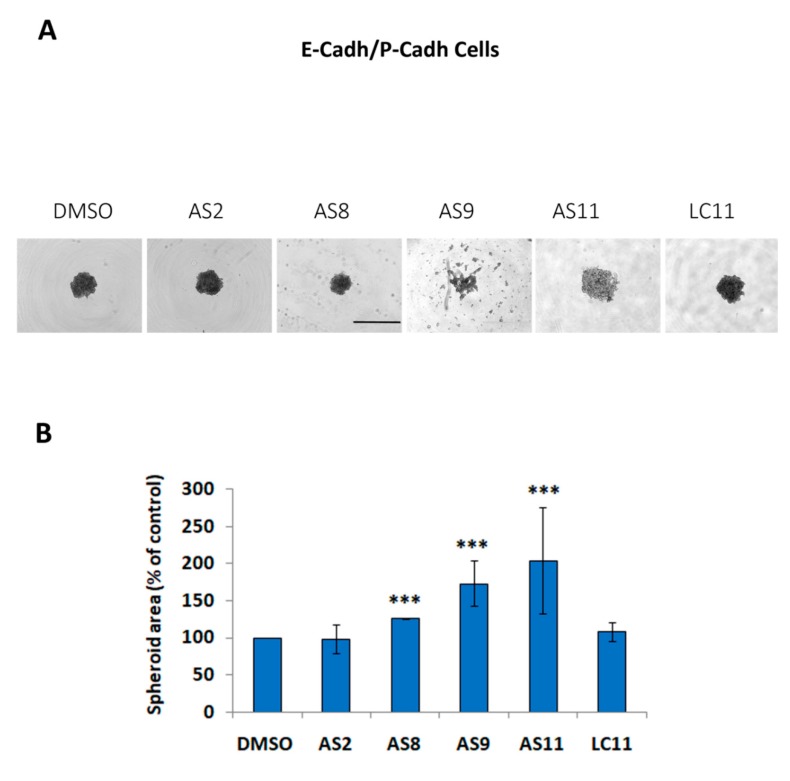
AS11, AS9, and, to a lesser extent, AS8 regulate cell–cell adhesion. BxPC-3 E-cadh/P-cadh cells were incubated in the presence of either 0.1% DMSO or 0.05 mM AS2, AS8, AS9, AS11, or LC11 and allowed to form spheroids in suspension for 24 h. (**A**) Representative pictures of spheroids. Scale bar: 1000 μm. (**B**) The spheroid area was measured by phase-contrast microscopy and analyzed by ImageJ. Values represent the mean of two experiments performed in octuplet. A *p* < 0.05 was considered statistically significant and is indicated by ‘***’ when *p* < 0.001. See the Materials and Methods section for details on the statistical analysis.

To avoid solubility issues, we carried out cell–cell adhesion assays at an inhibitor concentration of 0.05 mM. As shown in Figure 2A, at this concentration only three molecules (AS11, AS9, and, to a lesser extent, AS8) retained anti-cell–cell adhesion activity. Quantification of the spheroid areas confirmed that cell treatment with AS9 and AS11 (and, to a lesser extent, with AS8) promoted formation of spheroids that were significantly less compact than when cells were treated with DMSO (Figure 2B).

**Figure 3:**
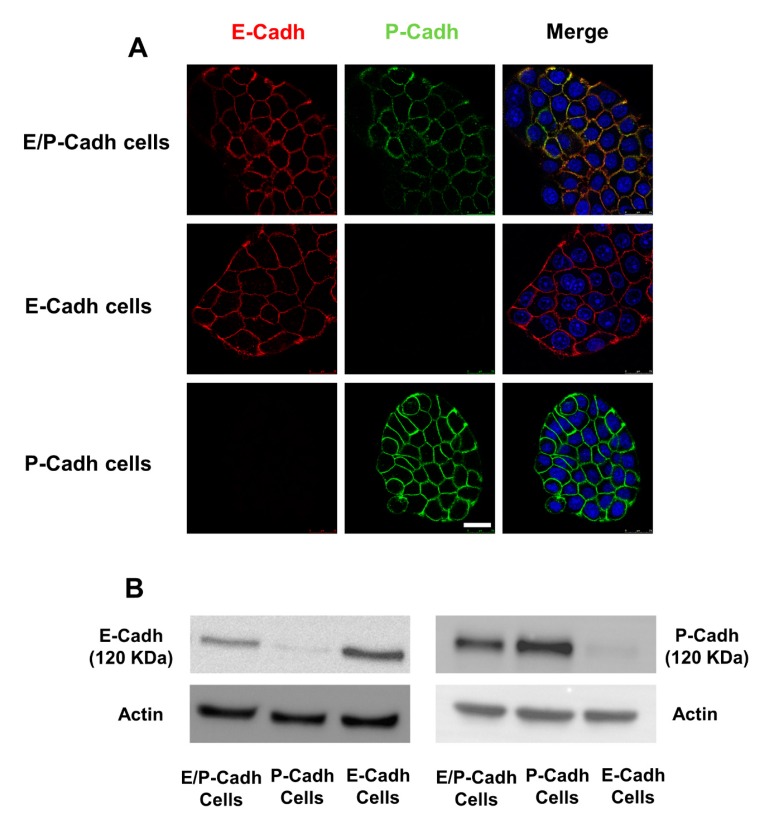
Cell surface cadherin extinction was assessed by both immunofluorescence and western blot. (**A**) Using RNA interference, E- and P-cadherin were stably knocked down in human pancreatic tumor BxPC-3 cells expressing both cadherins (see the Materials and Methods section for experimental details). Images were captured and analyzed using a SP5 Leica confocal microscope equipped with LAS AF Lite software. Scale bar: 25 μm. (**B**) BxPC-3 E-cadh/P-cadh, BxPC-3 E-cadh, and BxPC-3 P-cadh cells were lysed, and the expressions of both P-cadherin and E-cadherin were detected by western blot.

It was previously demonstrated that in BxPC-3 E-cadh/P-cadh, both cadherins participate in cell–cell adhesion, although E-cadherin is the major player in cell–cell adhesion [32]. To determine which of the two cadherins is impacted by the compounds, we stably knocked down P-cadherin or E-cadherin in the BxPC-3 cell line by RNA interference; therefore, we performed a cell–cell adhesion assay using BxPC-3 E-cadh or BxPC-3 P-cadh as cell models (Figure 3).

**Figure 4:**
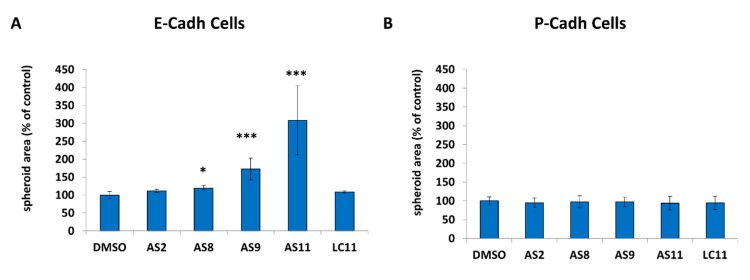
Both AS11 and AS9 target E-cadherin. BxPC-3 E-cadh cells (P-cadherin depletion) (**A**) and BxPC-3 P-cadh cells (E-cadherin depletion) (**B**) were incubated in the presence of 0.05 mM (E-cadh cells) or 0.1 mM (P-cadh cells) AS2, AS8, AS9, AS11, or LC11 and allowed to aggregate in suspension for 24 h. The spheroid area was measured by phase-contrast microscopy and analyzed by ImageJ. A *p* < 0.05 was considered statistically significant and is indicated by ‘***’ when *p* < 0.001 and ‘*’ when *p* < 0.05. See the Materials and Methods section for details on the statistical analysis.

**Figure 5:**
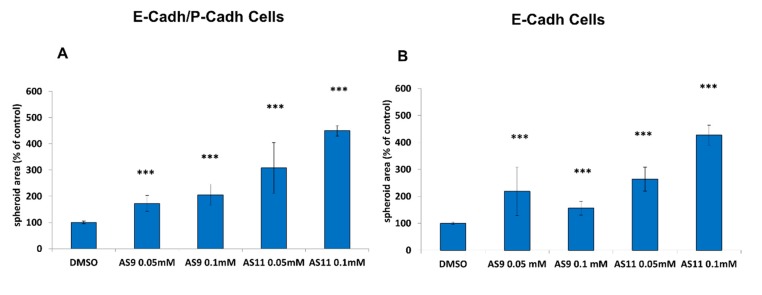
AS11 is more potent than AS9 to impair cell–cell interaction. BxPC-3 E-cadh/P-cadh cells (no cadherin depletion) (**A**) and BxPC-3 E-cadh cells (P-cadherin depletion) (**B**) were incubated in the presence of AS9 or AS11 at various concentrations and allowed to form spheroids in suspension for 24 h. The spheroid area was measured by phase-contrast microscopy and analyzed by ImageJ. A *p* < 0.05 was considered statistically significant and is indicated by ‘***’ when *p* < 0.001. See the Materials and Methods section for details on the statistical analysis.

**Figure 6:**
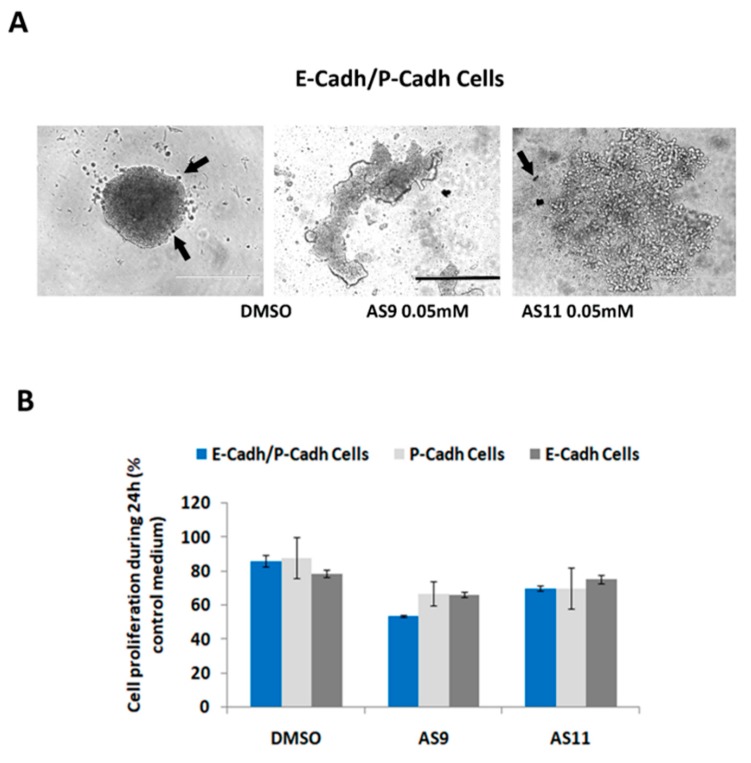
(**A**) BxPC-3 E-cadh/P-cadh cells were treated with 0.1% DMSO or 0.05 mM of inhibitor and allowed to form aggregates for 24 h. Cell viability was then assessed by a trypan blue exclusion test. Black arrows indicate dead cells. Scale bar: 400 μm (**B**) BxPC-3 E-cadh/P-cadh, BxPC-3 E-cadh, and BxPC-3 P-cadh cells were seeded at a density of 4000 cells per well in 96-well plates. Twenty-four hours after plating, cells were further incubated for another 24 h with 0.1% DMSO or with 0.05 mM of AS9 or AS11. Cell numbers were analysed using thiazolyl blue tetrazolium bromide (Sigma) staining according to the manufacturer’s instructions.

Both AS09 and AS11 efficiently impaired BxPC-3 E-cadh cell–cell adhesion at 0.05 mM (Figure 4A and Figure 5B), while at the same concentration they had no effect on cells expressing only P-cadh (Figure 4B). It should be noted that both AS9 and AS11 influenced cell–cell adhesion without affecting cell viability, as observed by the trypan blue exclusion assay (Figure 6A). We also observed that 24 h incubation with these compounds slightly reduced the cell number when cell models were cultured on plastic (Figure 6B). This effect, which may reflect modulation of cell proliferation, was independent of cadherin expression since it was observed regardless of the cell model used.

Quite clearly, AS9 affected BxPC-3 E-cadh cell aggregation less efficiently than AS11 for whichever concentration was used (Figure 5). Indeed, while both AS9 and AS11 showed a clear dose–response relationship when tested at different concentrations (0.05 and 0.1 mM), based on the area of the spheroids formed by both BxPC-3 E-cadh/P-cadh and BxPC-3 E-cadh cells after 24 h of incubation with the two compounds, AS11 showed greater potency than AS9.

**Figure 7:**
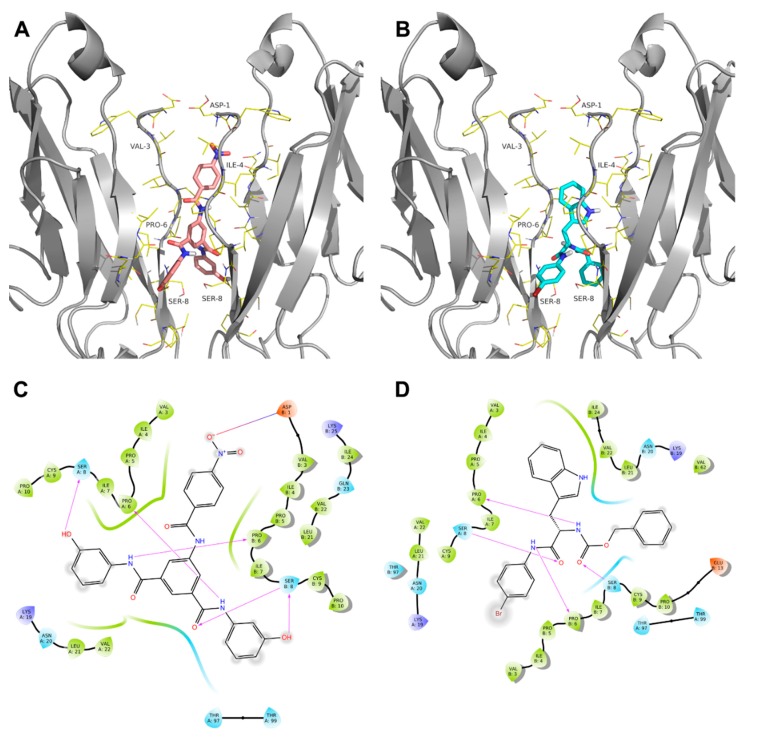
3D representation of the theoretical binding mode for AS9 (**A**) and AS11 (**B**). 2D ligand interaction diagram of the theoretical binding mode for AS9 (**C**) and AS11 (**D**). Hydrogen bond interactions are shown as pink arrows. Positive and negative charged amino acids are represented in blue and red, respectively. Residues involved in hydrophobic or polar interactions are shown in green and light blue, respectively. Ligand-exposed fractions are indicated as a gray, circular shadow.

These results strongly suggest that AS11 and, to a lower extent, AS9 blocked E-cadherin-dependent cell–cell adhesion. Interestingly, all our numerous attempts to crystallize either AS9 or AS11 in complex with E-cadherin following the same approach used for FR159 failed, most likely because of the much higher potency of these two compounds relative to FR159, which makes them incompatible with the formation of the stable hydrophobic pocket where FR159 was found to bind the E-cadherin X-dimer [27]. The theoretical binding mode for the two most potent inhibitors identified in this study is shown in Figure 7.

### 2.2. Cell Invasion

**Figure 8:**
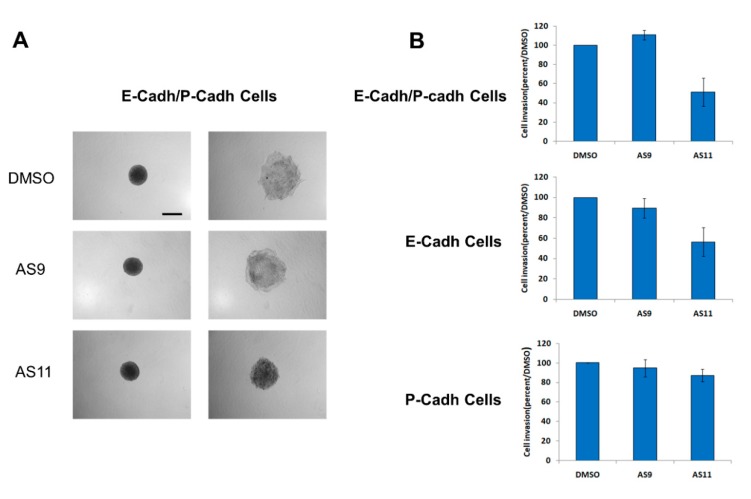
AS11, but not AS9, regulates cell invasion. BxPC-3 E-cadh/P-cadh cells (no cadherin depletion), BxPC-3 E-cadh cells (P-cadherin depletion), and BxPC-3 P-cadh cells (E-cadherin depletion) were allowed to form spheroids for 72 h. Spheroids were then embedded in type I collagen. After embedding, followed by a 24 h incubation in the presence of either 0.2% DMSO or 0.1 mM AS9 or AS11, the spheroid area was observed by phase contrast microscopy (**A**). Scale Bar: 500 μm. (**B**): the spheroid area was measured using ImageJ. Results are expressed as the percentage of invasion of treated cells compared to control cells.

Regulation of cadherin-mediated cell–cell adhesion is known to modulate cancer cell invasion. For instance, both E-cadherin and P-cadherin are involved in pancreatic cancer cell invasion [32]. Hence, targeting cadherins in pancreatic cancer may constitute an effective therapeutic intervention. From this perspective, we set out to analyze the impact of both AS9 and AS11 compounds on the invasive capacity of the three human pancreatic cell models. BxPC-3 E-cadh/P-cadh organized in spheroids invaded a 3D type I collagen gel (Figure 8A). Interestingly, AS11, but not AS9, was able to decrease the invasive capacity of E-cadherin-expressing cells, i.e., both E-cadh/P-cadh and E-cadh cells (Figure 6B). However, neither AS9 nor AS11 decreased BxPC-3 P-cadh cell invasion (Figure 8B).

**Figure 9:**
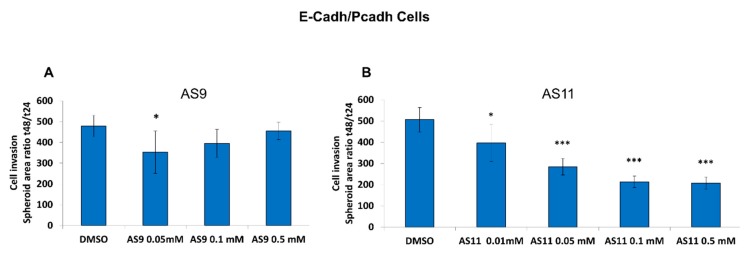
Spheroid area ratio t48/t24 for AS9 and AS11. BxPC-3 E-cadh/P-cadh cells (no cadherin depletion) were allowed to form spheroids for 72 h. Spheroids were then embedded in type I collagen and incubated for 48 h in the presence of either 0.1% DMSO or AS9 (**A**) or AS11 (**B**) concentrations ranging from 0.01 to 0.5 mM. The spheroid area was observed by phase-contrast microscopy and measured after 24 and 48 h incubation. See the Materials and Methods section for details on the statistical analysis. A *p* < 0.05 was considered statistically significant and is indicated by ‘***’ when *p* < 0.001 and ‘*’ when *p* < 0.05. See the Materials and Methods section for details on the statistical analysis.

This blockage of cell invasion effect could be observed for a concentration as low as 0.05 mM when AS11 was used (Figure 9). Taken together, these results confirm that AS11 selectively targets E-cadherin rather than P-cadherin. Since depletion of one of the both cadherins expressed leads to a decrease in cell invasion, this indicates that AS11 could be considered as an anti-invasive compound for pancreatic cancer cells.

## 3. Discussion

Owing to its N-cadherin inhibition properties, the cyclic peptide ADH-1 has long been studied in clinical trials with cancer patients [29,30,31]. However, while its role in modulating N-cadherin-mediated cell–cell adhesion has been ascertained [1], no information regarding its actual binding mode and inhibition mechanism has ever been derived, thus posing a serious limitation to the design of more effective cadherin homodimerization inhibitors. The recent crystal structure of the human E-cadherin-EC1EC2 fragment in complex with the peptidomimetic compound FR159 [27] provides, for the first time, clear experimental evidence of a possible strategy for cadherin homo-dimerization inhibition. As such, it also provides a unique opportunity to discover novel and more effective cadherin inhibitors via a structure-based drug discovery approach that has never been possible before. In that structure, the FR159 ligand binds across the hydrophobic pocket that forms, transiently, at the level of the two neighboring EC1 domains when the protein reaches the weakly adhesive X-dimer conformation as it moves along its dimerization trajectory (Figure 1). While that pocket, as a result of the cadherin dynamic behavior, may not necessarily represent the only druggable cadherin surface to be exploited for inhibition purposes, it is, however, a region where suitable ligands can clearly interfere with the cadherin homo-dimerization process and prevent the system to proceed towards the final strand-swap dimer conformation. Hence, based on that crystal structure, we took a virtual screening approach to identify commercially available drug-like molecules that may act similarly to FR159, and we tested a number of them in human pancreatic tumor BxPC-3 cells expressing both E-cadherin and P-cadherin, which would allow us to probe, selectively, modulation of cadherin function.

Indeed, we used RNA interference to silence each of the two cadherins individually, and we compared the efficiency of the VS-derived compounds in affecting the area of the spheroids formed by BxPC-3 E-cadh/P-cadh, BxPC-3 E-cadh, and BxPC-3 P-cadh cells. By this approach, we were able to identify two compounds (AS9 and AS11) that were able to inhibit cadherin-mediated cell–cell adhesion at a 50 µM concentration, and without exhibiting any cytotoxic effect, by acting selectively on E-cadherin and not on P-cadherin. Moreover, at the same concentration, AS11 was also found to block the invasive capacity of E-cadherin-expressing cells, while no limitation of the invasive capacity of P-cadherin-expressing cells could be observed. Given the specificity of interaction of these two compounds with E-cadherin, their lack of cytotoxicity and their greater than 20-fold improvement in potency compared to ADH-1, we believe that our approach has led to the identification of selective E-cadherin modulators with potential pharmaceutical profiles that can also serve as new starting points for further rounds of optimization.

Moreover, given the structural similarity between different classical cadherins, it is conceivable that a similar structure-based approach may lead to the identification of inhibitors that are selective for other cadherin family members. For instance, as both N- and VE-cadherin are involved in blood vessel formation [33], the development of selective inhibitors against these two targets would likely provide anti-angiogenesis tools to impair tumor vasculature stability. Sprouting of new vessels from the surrounding vasculature is, in fact, a requirement for the growth of solid tumors [34]. Indeed, various monoclonal antibodies against VE-cadherin have been shown to effectively destabilize tumor vasculature [35,36,37]. Likewise, anti-N-cadherin antibodies have been shown to cause microvessel bleeding [38].

Another interesting pharmaceutical target is cadherin-11, a mesenchymal cadherin that is expressed in many tissues such as skin and lung, but it is mainly found in osteoblasts and in synovial fibroblasts [39]. Cadherin-11 plays a crucial role in the development of the synovium, a layer of cells that lines the joints and provides lubrication for the cartilage [40]. As fibroblasts are able to produce cytokines, chemokines, and other proinflammatory molecules, they can play a major role in inflammatory disorders. For instance, in rheumatoid arthritis, the synovium is the main site of inflammation and transforms into a pannus tissue that invades and damages the cartilage [41]. Studies using monoclonal antibodies against cadherin-11 in mouse models of inflammatory arthritis have demonstrated that cadherin-11 inhibition reduces cytokine production by synovial fibroblasts, thus contrasting their pathological behavior [40].

Finally, the development of selective cadherin inhibitors may allow contrasting cancer cell metastasis. Indeed, loss of E-cadherin expression occurring in cancer cells undergoing EMT often correlates with aberrant N-cadherin and cadherin-11 up-regulation. This phenomenon, commonly referred to as a cadherin switch, facilitates the development of metastases in other tissues, such as the brain and the bones, that constitutively express those cadherins. As for the risk of inducing an EMT effect when using E-cadherin inhibitors, it should be stressed that E-cadherin-mediated cell–cell adhesion may not necessarily need to be completely abolished in pathological states. Rather, a subtle modulation of cadherin-mediated cell–cell adhesion may provide the desired effect on tumor compactness without triggering a cadherin switch.

Clearly, the data shown herein demonstrate the validity and the importance of the previously determined crystal structure of the complex between the E-cadherin-EC1EC2 fragment and the FR159 peptidomimetic compound. This unique complex structure led to the identification of a previously undetected druggable cadherin pocket that forms when the classical cadherin reaches the intermediate, adhesive, X-dimer conformation. Indeed, the crystal structure of the E-cadherin-EC1EC2-FR159 complex, which served as the starting point for this study, provided, for the first time, clear evidence of a viable inhibitory mechanism. This paves the way for future structure-based drug development studies aimed at the selective modulation of cadherin-mediated cell–cell adhesion in different pathological settings and with different cadherin targets.

## 4. Materials and Methods

### 4.1. Database Preparation

Asinex (http://www.asinex.com/) and LifeChemicals (http://www.lifechemicals.com/) databases were downloaded as .smi files from ZINC [DOI: 10.1021/ci3001277] and joined together.

The .smi files were initially converted to .mae with the “smiles_to_mae” script available in the Schrodinger suite 2015-4 [Schrödinger, LLC, New York, NY, 2015]. The 2D structures were converted into the corresponding 3D structures, and all tautomers and enantiomers were generated at pH 7.0 ± 1.0 using the LigPrep tool with Epik and OPLS2005 as a force field.

PAINS, compounds with more than two chiral centers, and all the duplicates were removed using the “ligfilter” script of the Schrodinger suite.

### 4.2. Protein Preparation

Since the adhesion arm was truncated in the X-dimer conformation of human E-cadherin (PDB code: 4ZTE), the missing residues were reconstructed from the coordinates extracted by a molecular dynamics (MD) trajectory of the complete X-dimer taken from PDB code 1FF5 [42]. In particular, the EC1EC2 ectodomain fragment (residues 1–213) was employed after removing the extra N-terminal methionine and the C-terminal 214–218 residues. The *tleap* module of AmberTools 14 was used for system setup [43]. The protein was solvated in a truncated octahedral TIP3P box with a buffer of at least 14 Å in every dimension, and the net charge was neutralized by adding counterions [44,45]. The protein was treated with an Amber ff99SP-ildn force field together with reoptimized ω-dihedrals [46,47]. Moreover, the three calcium ions located at the EC1EC2 interface of each monomer were modeled through the octahedral dummy model proposed by Duarte et al. [48]. The size of the final system was more than 230,000 atoms.

MD simulations were carried out in the canonical ensemble using NAMD-2.10 [49]. A nominal temperature of 300 K was maintained by performing Langevin dynamics with a damping coefficient of 5 ps^−1^. A multiple time-stepping scheme was used, by evaluating short-ranged interactions every 2 fs, while full electrostatics were computed every 4 fs. Nonbonded interactions were evaluated with a cutoff threshold of 10 Å. The particle mesh Ewald (PME) method [50], with a number of grid points equal to 120 along each dimension, was used to compute long-range electrostatic interactions. After equilibrating the system, the dynamics were extended until a frame very close to human E-cadherin could be identified (5 ns). Finally, a model of human E-cadherin was built using the coordinates of the adhesion arms extracted from the final MD frame, and it was added to the X-dimer conformation of the protein as solved in the PDB code 4ZTE.

The model thus obtained was processed with the Schrodinger “Protein Preparation Wizard” tool. The H-bonds were assigned with PROPKA at pH 7.0, and a restrained minimization was performed to relieve steric clashes (we used a convergence criterion of 0.30 Å of root-mean-square deviation (RMSD) for the heavy atoms with respect to the initial structure and OPLS2005 as force field).

### 4.3. High-Throughput Docking (HTPD)

The database of commercially available compounds was docked into the prepared protein (centered on the position of the peptidomimetic inhibitor FR159 (PDB code: 4ZTE), with the option “dock ligands similar in size to the workspace ligand”) using the Glide software with standard precision (SP).

Only the top 1000 scored compounds were visually inspected, and 200 of them were selected. The selected compounds were clustered on the basis of the tanimoto similarity index for the “Molprint 2D” fingerprint calculated with Canvas at 64-bit precision. Finally, 15 compounds were tested experimentally.

### 4.4. Ligands

All ligands were purchased from Asinex or Life Chemicals Inc. and had a purity of at least 95%. The molecules were dissolved in 100% DMSO and used without further purification. Subsequent dilutions in aqueous buffer were performed for biological testing.

### 4.5. Cell Models

The human pancreatic BxPC-3 cell line was routinely cultured in DMEM/10% fetal calf serum (FCS) and authenticated using short tandem repeat (STR) profiling (ATCC). Cells were cultured in the laboratory for no more than 10 passages and were tested for *Mycoplasma* every 3 weeks. BxPC-3 cells were used as a model system since these cells express high levels of both E-cadherin and P-cadherin at cell–cell contacts [32]. E- and P-cadherin were stably knocked down in the BxPC-3 cell line by RNA interference using mission shRNA lentiviral transduction particles (Sigma, St Quentin Fallavier, France) as previously described [32]. The generated stable cell lines were called E-cadh/P-cadh (no cadherin depletion), E-cadh (P-cadherin depletion), and P-cadh (E-cadherin depletion). Cell surface cadherin extinction was assessed by both immunofluorescence and western blot (Figure 3). For immunofluorescence detection, BxPC-3 E-cadh/P-cadh, BxPC-3 E-cadh, and BxPC-3 P-cadh cells were plated on glass coverslips then fixed for 20 min with 2% formaldehyde in PBS. Thereafter, cells were first permeabilized with 0.1% saponin in PBS for 20 min and then blocked for 30 min in PBS containing 4% (*w/v*) BSA. E-cadherin and P-cadherin were sequentially detected by incubation with mouse HECD-1 mAb (Takara, Saint-Germain-en Laye, France) and rabbit Ab (2130 s, Cell signaling Technology, Saint-Quentin-en-Yvelines, France), respectively. After three washes, samples were incubated for 1 h with Alexa Fluor 488- and 594-conjugated goat Ig (20 μg mL^−1^) and raised against mouse and rabbit Igs, respectively. Samples were then washed and mounted in ProLong Gold (Invitrogen, Illkirch, France). Images were captured and analyzed using a SP5 Leica confocal microscope equipped with LAS AF Lite software.

### 4.6. Cell–Cell Adhesion Assay

A spheroid formation assay was used to investigate the effect of inhibitors on cadherin-mediated cell–cell adhesion properties. Isolated cells were seeded onto U bottom untreated tissue culture 96-well plates at a density of 5000 cells per well in 100 μL DMEM containing 10% FCS and 0.24% methylcellulose in the presence or absence of inhibitors. Spheroids were grown for 24 h, and pictures were taken using an Olympus microscope (Objective 4X). The size of the spheroids was quantified by measuring the area occupied by cells using image J software (rsb.info.nih.gov/ij/). Each tested condition was done in dodecaplicate repeated 2 or 3 times.

### 4.7. D-Invasion Assay

Cells were cultured for 72 h as described above to obtain compact spheroids. The latter were embedded into 1.6 mg/mL of bovine collagen type I matrix (Advanced Biomatrix), diluted in 0.12% methylcellulose, and covered with serum-free medium in the presence or absence of inhibitors. Spheroids were then incubated for 24 h in a temperature- and CO_2_-controlled chamber. Images were taken using an Olympus microscope (Objective 4X). The size of the spheroids was quantified by measuring the area occupied by cells using ImageJ software (rsb.info.nih.gov/ij/). Each tested condition was done in sextuplet repeated 3 times.

### 4.8. Statistical Analysis

Data are presented as the mean ± S.D. for three independent experiments performed in triplicate. Comparison between the two conditions was made using the Mann–Whitney test. A *p* < 0.05 was considered statistically significant in all analyses and is indicated by ‘***’ when *p* < 0.001, ‘**’ when *p* < 0.01, and ‘*’ when *p* < 0.05.

## 5. Conclusions

Cadherins are highly dynamic systems that undergo major conformational changes throughout their entire dimerization trajectory, which features a number of transient and reversible intermediate states. Some of these states, such as the well-characterized X-dimer conformation, are adhesive in nature. Therefore, homo-dimerization occurs stepwise and begins to provide adhesive force well before reaching the endpoint of the dimerization process (i.e., strand-swap conformation). Owing to this dynamic behavior and to the existence of multiple adhesive states, efficient modulators or inhibitors of cadherin-mediated cell–cell adhesion are difficult to develop. Based on the first and only crystal structure available of a cadherin extracellular fragment in complex with a small molecule inhibitor (FR159), we conducted a virtual screening analysis of databases of drug-like molecules to identify more potent and specific modulators of cadherin-mediated cell–cell adhesion. By cell–cell adhesion assay analysis, we identified two compounds (AS11 and, to a lesser extent, AS9) that inhibit E-cadherin dimerization and dissociate cellular aggregates at 50 μM. This shows that the potency of AS11 is 20-fold higher than that of FR159, which is, in turn, substantially higher than the potency of ADH-1, the only cadherin inhibitor that, to date, has been tested in clinical trials. Finally, by performing cell invasion assays, we have also shown that at 0.1 mM concentration, AS11 interacts with E-cadherin to modulate cell invasion, while no significant effect on cell invasion can be observed on cells expressing P-cadherin only.

The functional data shown herein further validate the druggable interface formed by two cadherin proteins in the weakly adhesive X-dimer conformation previously identified by X-ray crystallography. Therefore, they confirm that a structure-based approach to the identification of effective cell–cell junction modulators to be used as antiangiogenic drugs for the treatment of cadherin-expressing solid tumors, as anti-inflammatory drugs against RA or as potential pharmaceutical excipients to facilitate drug delivery across biological barriers is now possible.

## Figures and Tables

**Table 1:** List of the tested compounds.

Company (ID#)	Internal Code	Chemical Structure
Asinex (AEM14687298)	AS2	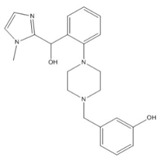
Asinex (BAS00093476)	AS8	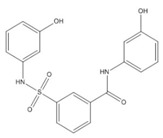
Asinex (BAS00132635)	AS9	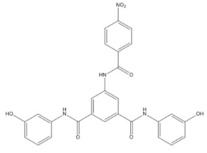
Asinex (BAS00602705)	AS11	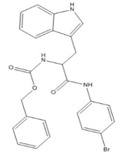
Life Chemicals (F2762-0527)	LC11	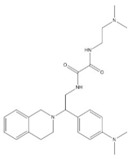

## Abbreviations

E-Cadh	E-cadherin
P-Cadh	P-cadherin
EMT	Epithelial-to-Mesenchymal Transition
DMSO	Dimethyl sulfoxide
DMEM	Dulbecco’s Modified Eagle Medium
PBS	Phosphate-buffered saline
BSA	Bovine serum albumin
EOC	Epithelial Ovarian Cancer
FCS	Fetal Calf Serum
STR	Short Tandem Repeats
VS	Virtual screening
shRNA	Short hairpin RNA
MD	Molecular Dynamics
PME	Particle mesh Ewald
RMSD	Root-Mean-Square Deviation
HTPD	High Throughput Docking
PDB	Protein Data Bank
